# The meaning of the 'impact factor' in the case of an open-access journal

**DOI:** 10.1186/1750-0680-6-1

**Published:** 2011-03-02

**Authors:** Georgii A Alexandrov

**Affiliations:** 1 Institute of Atmospheric Physics, Russian Academy of Sciences, Pyzhevsky 3, Moscow, Russia

## Abstract

The dominant model of journal evaluation emerged at the time when there were no open-access journals, and nobody has assessed yet whether this model is able to cope with this modern reality. This commentary attempts to fill the gaps in the common understanding of the role that 'impact factor' should play in evaluation of open-access journals.

## Introduction

Fifty years ago, when computers were a rarity, librarians used index cards to keep records of library holdings. The cards were arranged into an author index and a subject index. To find publications related to the subject of his or her research, a researcher went to the subject index, drew the box with the cards on the subject, look at the long row of cards, and did not know where to start. Since the cards were ordered alphabetically, the seminal works on the subject were lost in the mass of less important publications.

Pointing out that subject indexes were "limited in their attempt to provide an ideal key to the literature", Eugene Garfield proposed, in 1955, a new bibliographic tool -- a "citation index"[1]. The underlying idea of this tool may be formulated as follows. A seminal work could be an ideal key to the literature on the subject to which it gives the birth, if all the references to this work were to be listed on its card (see details in Appendix).

In 1960, Garfield founded the Institute for Scientific Information (ISI), and started a bibliographic indexing service-the Scientific Citation Index, which has been continued since 1992 by the Thomson Reuters Corporation.

In 1975, ISI began to publish Journal Citation Reports (JCR), which became later "the recognized authority for evaluating journals" [2]. The logic of journal evaluation was developed by Garfield [3] at the time when there were no open-access journals, and nobody has assessed yet whether the logic of counting citations is able to cope with this modern reality.

## Discussion

The JCR summarizes citation data and delivers detailed reports that are supposed to be helpful for librarians, authors, editors, publishers and administrators [2]. The most known metric provided by the JCR is the journal impact factor: the ratio of the current year citations to the source items published in the journal during the previous two years. The following four examples are to show how it may help librarians, authors, editors and publishers:

(1) If a library is subscribed for two journals with roughly the same title, and cannot continue subscription to both of them, then the journal impact factor has a certain meaning for the librarian: it gives an argument for choosing the journal that should be removed from the library collection.

(2) If the subject of an article falls within the scope of two journals, then the journal impact factor has a certain meaning for the article's author: it gives an argument for choosing the journal where to submit the article.

(3) If the impact of a journal is higher than those of other journals in the same field of science, then the journal impact factor has a certain meaning for the journal's editor: it gives an argument for convincing a highly-cited scientist to give an article for the journal.

(4) If the impact of a journal is higher than those of other journals in the same field of science, then the journal impact factor has a certain meaning for the journal's publisher: it gives an argument for convincing a librarian to subscribe to the journal.

Should the meaning of the impact factor remain the same in the case of an open-access journal? Obviously, the impact factor of an open-access journal should not have a practical meaning for a librarian, as the libraries need not subscribe to the open-access journals. As to the other examples, the answer is not so obvious.

Open-access journals have a number of features that make them different from the subscription-based journals. The most important of them is that the readership of an open-access journal is not restricted by subscription. This is a huge advantage in sense of dissemination of research results: each article may receive as wide readership as it deserves. It does not matter whether the journal impact factor is high or low: in any case, the articles published in an open-access journal are not less available for a broad readership than the articles published in Science or Nature.

Another feature is that open-access journals are integrated to the information environment that is formed by search engines, social networks and other tools for harnessing internet power for quick "association of ideas". All these tools provide direct access to the articles. In this information environment, we read articles, not journals. To read an article, we need not open a journal and read the contents. We even need not read the journal title. Thus, again, it does not matter whether the journal impact factor is high or low: a search engine will find the article, and its position in the search list will depend on its relevance to the search query.

In other words, the impact factor of an open-access journal should not have a significant meaning for an author, if the author strives for dissemination of research results.

It would be credulous to believe, however, that dissemination of research results is the only purpose of a scientific publication. An article published in a peer-reviewed journal is a sign of scholarly recognition. Moreover, a publication in a high-rank journal is not merely a sign of recognition -- it is a sign of distinction. Hence, the rank of the journal, whatever it means, is important. Having no rank, received from JCR (or other relevant "authority"), an open-access journal would not attract the author who need such a sign of distinction as a publication in a high-rank journal.

Shall open-access journals compete for the authors who seek distinction? If we are all agree that subscription-based journals should not be extinct, it would be prudent to reserve them for distinctive authors. Open-access journals have been introduced under the flag of research dissemination, and may thrive under this flag.

Of course, we need some metrics to assess usability of a journal for disseminating research results. Bibliographic metrics are not very suitable for this purpose, as the number of citations received by an article depends on the quality of the article. It would be better to develop the metrics that would be less sensitive to the bibliographic quality of a journal and more sensitive to its quality as a medium for conveying research results. Since the websites of the journals published by BioMed Central look like web-portals to corresponding fields of science, it might be reasonable to use the metrics that are proposed for evaluation of web-portals [4].

## Conclusions

The brief analysis of the role that 'impact factor' should play in evaluation of open-access journals shows that this issue needs a critical consideration along the following four points:

(1) The open-access business model has been introduced for solving the problem that could not be solved by means of the subscription-based business model -- that is, the problem of how to give *every *research article as wide readership as it deserves.

(2) The value of an open-access journal as a tool for communicating research results may be more important than the bibliographic value of the journal as a collection of research articles.

(3) The use of the journal impact factor for evaluating an open-access journal may obscure the role of the journal as an information and communication tool.

(4) The journal impact factor should not play a crucial role in evaluation of open-access journals, if we all agree that the open-access journals are not to replace the subscription-based journals.

It is worth noting here that CBM hopes to start reporting its impact factor in the future like many other journals published by BioMed Central. Meanwhile, authors and readers may retrieve bibliographic indicators of CBM impact from Scopus Journal Analyzer [5], and related bibliometric services [6-8]. They also may find at the journal website the statistics of accesses that gives some impression about the size of "non-citing" readership, which is ignored by bibliographic indicators, but which is of certain importance for a journal that publish articles on policy relevant aspects of global carbon cycle.

## Appendix. CBM as a starting point to a literature search

Proposing a new bibliographic tool, "citation index", Garfield argued that any comprehensive index to the literature of science may provide only a better starting point for a literature search than the one provided in the selective indexes. He wrote, "Proponents of classified indexes may suggest that classification is the solution to this problem, but this no means the case" [1]. The new bibliographic tool was "to span the gap between the subject approach of those who create documents -- that is, authors -- and the subject approach of the scientist who seeks information".

In the later work [9], Garfield specified more clearly the major difference between subject indexes and citation indexes. The user of a citation index must know some work associated with the subject of his or her research. "If the user does not know of a previous work on the subject he must find one through a book, an encyclopedia, or a colleague." That is to say, the user should know key works, instead of keywords.

The publications that cite the same key work form a well defined class. They are all somehow associated with the subject of this key work. Besides, the publications citing these publications also belong to this class. Hence, quite a large bibliography may be created starting from a single key work.

**Figure 1:**
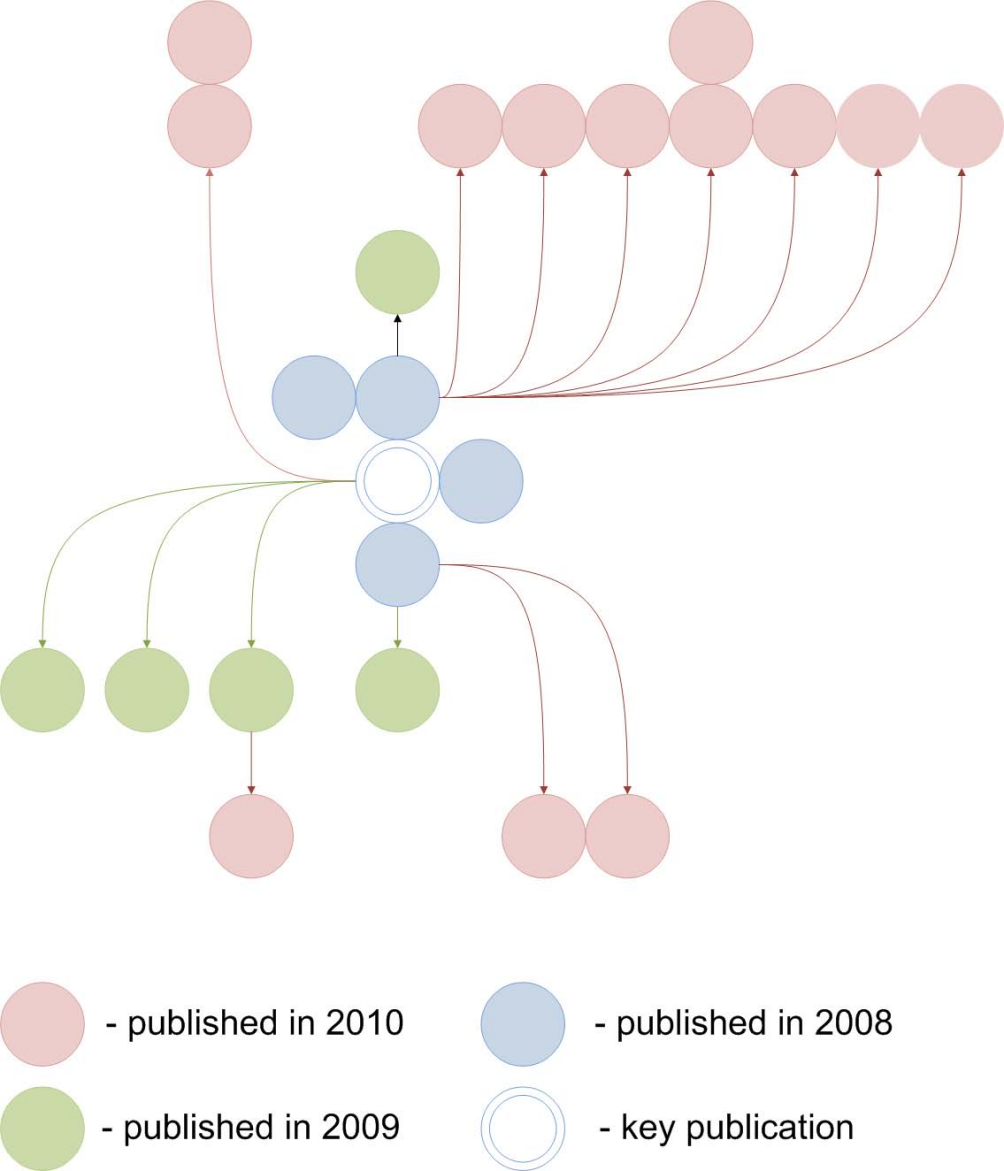
**Key works as keywords**. The structure of the bibliography that could be created starting from a CBM article.

The Figure 1 shows the structure of a bibliography that could be created starting from a CBM article and using the algorithm demonstrated in the Additional file 1. This bibliography consists of 22 entries. Among them, 8 first-order citations, that is citations to this key work, 13 second-order citations, that is, citations to the works citing the works that cite this key work, and 1 third-order citation.

Some articles published in CBM received more than 30 first-order citations. They may be used to generate bibliographies consisting of hundreds of entries. However, an extensive bibliography is not the purpose of every literature search. More than often, a literature search is to find a dozen of the most recent articles that somehow correspond to the completed research and hence could be cited to demonstrate the novelty and significance of the research result.

In the example considered above (Figure 1), 9 of 13 references to the articles published in the year of 2010 cite the most cited first-order citation. Hence, if the purpose of a literature search is to find a dozen of the most recent references, then 'citation surfing', that is, tracking only the most cited citations would be more reasonable than a comprehensive citation analysis.

'Citation surfing' may efficiently reduce the list of articles returned by citation analysis. Besides, not only articles but also the names of the editorial board members can be used as a starting point for 'citation surfing' (see Additional file 2). Another advantage of 'citation surfing' is that "it brings together material that would be never collated by the usual subject indexing"[1], reveals unexpected associations of ideas, and thus stimulates creative thinking.

In the earlier works of Garfield [1,9], citation analysis was promoted as a tool for communicating information. Now, it is promoted as a tool for evaluating information. Citation analysis has potential for both communication and evaluation. The obsession with the impact factor obscures the value of citation analysis as a tool for communicating information.

## Supplementary Material

Additional file 1 **A demonstration of how CBM can be used as a starting point to a literature search**. The file can be viewed by using Windows Media Player. Click here for file

Additional file 2 **A demonstration of how the names of editorial board members can be used as starting points for 'citation surfing'**. The file can be viewed by using Windows Media Player. Click here for file
